# Unraveling the Molecular Determinants of Manual Therapy: An Approach to Integrative Therapeutics for the Treatment of Fibromyalgia and Chronic Fatigue Syndrome/Myalgic Encephalomyelitis

**DOI:** 10.3390/ijms19092673

**Published:** 2018-09-09

**Authors:** José Andrés Espejo, María García-Escudero, Elisa Oltra

**Affiliations:** 1 School of Experimental Sciences, Universidad Católica de Valencia San Vicente Mártir, 46001 Valencia, Spain; joseandres.espejo@mail.ucv.es; 2 School of Physiotherapy, Universidad Católica de Valencia San Vicente Mártir, 46900 Valencia, Spain; maria.escudero@ucv.es; 3 School of Medicine, Universidad Católica de Valencia San Vicente Mártir, 46001 Valencia, Spain; 4 Unidad Mixta CIPF-UCV, Centro de Investigación Príncipe Felipe, 46012 Valencia, Spain

**Keywords:** fibromyalgia (FM), chronic fatigue syndrome/myalgic encephalomyelitis (CFS/ME), manual therapy (MT), clinical trials (CTs), integrative medicine, physiotherapy

## Abstract

Application of protocols without parameter standardization and appropriate controls has led manual therapy (MT) and other physiotherapy-based approaches to controversial outcomes. Thus, there is an urgency to carefully define standard protocols that elevate physiotherapy treatments to rigorous scientific demands. One way in which this can be achieved is by studying gene expression and physiological changes that associate to particular, parameter-controlled, treatments in animal models, and translating this knowledge to properly designed, objective, quantitatively-monitored clinical trials (CTs). Here, we propose a molecular physiotherapy approach (MPTA) requiring multidisciplinary teams, to uncover the scientific reasons behind the numerous reports that historically attribute health benefits to MT-treatments. The review focuses on the identification of MT-induced physiological and molecular responses that could be used for the treatment of fibromyalgia (FM) and chronic fatigue syndrome/myalgic encephalomyelitis (CFS/ME). The systemic effects associated to mechanical-load responses are considered of particular relevance, as they suggest that defined, low-pain anatomic areas can be selected for MT treatment and yet yield overall benefits, an aspect that might result in it being essential to treat FM. Additionally, MT can provide muscle conditioning to sedentary patients without demanding strenuous physical effort, which is particularly detrimental for CFS/ME patients, placing MT as a real option for integrative medicine programs to improve FM and CFS/ME.

## 1. Introduction

Fibromyalgia (FM), according to the International Classification of Diseases, Tenth Revision, Clinical Modification (ICD-10-CM) M79.7, including fibromyositis, fibrositis and myofibrositis, is described as a chronic disease of unknown origin, leading to low pain threshold, together with stiffness and tenderness of the muscles, often accompanied with general fatigue, sleep disturbances, headaches, and memory loss [1,2,3,4]. Similarly, chronic fatigue syndrome/myalgic encephalomyelitis (CFS/ME) (ICD-10-CM R53.82 or G93.3 if post-viral) is defined as an acquired complex multisystem disease with characteristic clinical features that include exercise-induced fatigue, post-exertional malaise (PEM)/symptom exacerbation, cognitive dysfunction, orthostatic intolerance, on-going flu-like symptoms, and unrefreshing sleep, in conjunction with others [5,6]. Often FM, and CFS/ME show overlapping symptoms as FM patients experience chronic fatigue, and CFS/ME suffers from muscle tenderness and pain; thus, some authors have posited they are part of the same somatic syndrome [7,8]. In support of this hypothesis a recent analysis by Natelson et al., reports increased ventricular cerebrospinal fluid lactate levels in patients of CFS/ME, FM or both, with respect to healthy participants [9]. However, differences across a number of clinical and biological parameters, such as PEM and autonomic function [10,11,12], hormone system unbalance [13,14], gene expression and cytokine profiles [15], and blood microRNA (miRNA) levels [16,17,18,19], suggest that the underlying pathophysiology in FM may differ from that of CFS/ME. 

Current pharmacological treatments for patients suffering from FM and/or CFS/ME are mainly directed to palliate some symptoms [20,21,22], as clinical trials (CTs) have failed to conclusively provide overall benefits, together with no associated harms. Some treatments, however, seem to support significant improvement for certain patient subgroups. This seems to be the case for the N-methyl-D-Aspartate (NMDA) antagonist memantine [23] or the dopamine 3 receptor agonist pramipexole [24] for the treatment of FM and the anti-CD20 antibody Rituximab directed to B-cell depletion [25] for treatment of CFS/ME. In this last case, caution is recommended as in vitro treatment of natural killer (NK) cells with the agent leads to significant decreases in NK lysing activity and a significant increase in cell degranulation, suggesting that Rituximab may be toxic for NK cells [26]. A more promising option for the treatment of CFS/ME is provided by the CTs using the Toll-like Receptor TLR-3 agonist rintatolimod (Poly I:C(12)U), which activates interferon-induced proteins, showing medically significant improvement in some cohorts of participating patients [22,27].

Alternative, non-pharmacological therapeutics, have also been extensively studied. Cognitive behavioral therapy (CBT) seems to lead to small benefits over control interventions in reducing pain, negative mood, and disability at the end of treatment, and at long-term follow-up in FM patients, as reported by 23 randomized controlled trials, including 1073 patients receiving CBT and 958 patients in control groups [28]. Although mindfulness meditation may be helpful in improving pain perception, it does not suffice for patients to recover their previous daily activity. 

Another non-pharmacological option is provided by gradual exercise therapy (GET). FM patients are able to engage in moderate to vigorous exercise; however, they experience difficulties performing and adhering to even moderate intensity regimes because of increased FM symptoms associated with exercise [29]. Benefits from CBT/GET therapy have also been reported for CFS/ME patients by other CTs (the PACE trials) including 160 participants per group, when compared to specialist medical care (SMC) alone or adaptive pacing therapy (APT) [30]. The authors of the PACE trials claim that the beneficial effects were maintained for one year at long-term follow-up, with a median of 2.5 years after randomization [31]. However, serious study design concerns have been raised by the scientific community regarding the inappropriate case definition of enrolled participants, scores that do not support significant improvement of fatigue and physical functioning at long-term, plus data indicative of subjective improvement by specialist medical care and APT to the same level as by CBT and GET, or without any additional therapies [32,33].

Even if exercise, which has shown promise in treating symptoms of centralized pain [34], could benefit FM and CFS/ME symptoms, the fact that exercise induces muscle pain and triggers exacerbated malaise in CFS/ME, makes this option unfit for these patients. Physiotherapy-based treatments, such as manual therapy (MT), on another end, might help providing exercise-like effects on treated tissues, as for example, increasing blood flow and/or increased muscle tone, without any physical activity demand from the patient, and thus, contrary to GET, should not compromise patient´s health. At the same time, and similarly to CBT, MT might engage patient´s mind into relaxation, boosting happiness.

To date, MT protocols, as most physiotherapeutic treatments, are poorly defined and yet, some CTs report benefits for massage therapy. For example, a systematic review and meta-analysis of randomized clinical trials (RCTs) by Li, Yuan et al., show that MT with duration ≥5 weeks leads to improvement in pain, anxiety, and depression in FM patients [35,36]. MT also seems to trigger positive effects on physical symptoms in CFS/ME, including depression, fatigue, pain, and insomnia [37,38,39], suggesting that MT could be used for therapeutic purposes by itself, or in combination with current symptomatic pharmacology as part of integrative medicine programs. 

The purpose of this review is to allocate a potential mechanism rationale for the effective treatment of FM and CFS/ME by MT. Future MT treatment protocols are expected to be capable of managing the symptoms that compromise daily activities in these patients and improve FM and CFS/ME health status in general. Towards this end, we have reviewed available preclinical mechanistic evidence from physical treatments of animal models, and identified molecular changes associated to MT parameters that could improve immune, cognitive and muscular dysfunctions on one side and alleviate pain on another (see Table 1), in an effort to build the initial basis for standardized therapeutic MT protocols to treat patients affected of FM and/or CFS/ME. 

It would be appropriate that future MT studies for the treatment of FM and CFS/ME are designed on the basis of quantitative objective traits associated to rigorously defined protocols. Validation of efficacy and optimization through CTs must demand close control of selected molecular or other disease-associated quantitative markers to objectively track individual responses of FM and CFS/ME patients to the received MT treatments. 

## 2. Molecular Determinants of MT: Lessons from Animal Models and Mimetic Devices

MT comprises a set of therapies based on the manual manipulation of joints and soft tissues, with the purpose of relieving pain, reducing inflammation, eliminating muscular contractures, increasing the range of motion (ROM), facilitating movement, etc. and ultimately, restoring health. It covers a very diverse range of techniques such as massage, muscular stretching, manipulations, and mobilizations among others.

Stretching protocols are amply used on body muscle tendon-units to gain flexibility and ROM of joints to improve or maintain health [47]. Due to our final purpose: treatment of main symptoms in FM and CFS/ME with standardized effective protocols, we will concentrate our attention on the available evidence for passive muscle stretching, defining it as a MT procedure that is effected by a professional physiotherapist on the patient. 

The other variant of MT that will be covered in this review is massage. Massage has been defined by Cafarelli and Flint, as a mechanical manipulation of body tissues with rhythmical pressure and stroking for the purpose of promoting health and well-being [48]. It is applied on soft tissues: skin, muscle, and conjunctive or connective tissue, sometimes with the help of mechanical or electrical devices to pursue various purposes, therapeutic included. There are different massage maneuvers (rubbing, friction, kneading, pressures, percussions, and vibrations) in relation to variables such as duration, frequency, repetitions, or pressure. Different benefits have been attributed to various massage maneuvers, for example, massage with moderate pressure seems to increase vagal tone and also be essential for stimulating subcutaneous mechanoreceptors that send pain relief signals to the brain and release de-stressing neurochemicals, such as serotonin and dopamine [49,50]. 

MT treatments are associated with mechano-transduction, a general biophysical process, by which cells are capable of sensing their physical environment and translating those cues into biochemical signals, such as shifts in intracellular calcium concentration, alteration of gene expression profiles, and the induction or repression of signaling pathways that finally lead to morphological and/or physiological changes [51,52], which may lead to therapeutic effects. 

Knowledge of the parameter-dependence that MT programs induce on treated tissues, at the molecular level, should, therefore, allow for the development of rigorous and standardized effective protocols (i.e. MPTA), providing health benefits to FM, CFS/ME and other patients. An initial step to acquire this knowledge on the MT-treated tissues involves evaluating profiles of gene expression of healthy tissues before and after particular carefully defined procedures. 

Methodological limitations apply to these studies with human subjects that are related not only to ethical concerns for sampling, but also to the application of the technique, such as the amount of load applied, and the frequency and duration of sessions. To overcome these limitations, preclinical animal trials with mimetic devices are being performed to identify molecules or biological patterns of interest in the target tissue to, optimally, translate the identified markers to a liquid biopsy test for human CT monitorization. With this final goal in mind, and a focus in particular disease problems, we proceed to summarize the molecular information of MT treatments in animal models that might be of relevance for the treatment of FM and CFS/ME (please use Table 1 as a guideline for this section).

### 2.1. The Neuroimmune Impact of MT

A group of researchers led by Dr. Dupont-Versteegden has objectively shown the effects of massage on healthy, unperturbed skeletal muscle on the modulation of key immune cells involved in the inflammatory response. For that purpose, the authors used Wistar rats (*N* = 24) and performed histological and microarray analysis on the tibialis anterior muscle after cyclic compressive loading (CCL). They used a custom-fabricated massage mimetic device to standardize and control the amount of load applied, the frequency and duration of sessions. 

The instrument consists of a spring-load mechanism allowing a cylinder (the load) to press and roll over a mass of tissue with an oscillating movement. Treatment for 30 min, once a day, for four consecutive days, using different loading conditions (1.4 to 11N), showed load-dependent molecular and cellular abundance changes of CD68 and CD163 positive subpopulations, with respect to sham loading controls. Moreover, load-independent changes were also evidenced on the non-CCL treated contralateral limb, indicating a systemic response of the massage-mimetic treatment [40]. From the 47% of the functional gene ontology clusters associating with immune response after CCL, the authors validated the chemokine (C-C motif) receptor CCR2, a critical regulator of skeletal muscle regeneration [53,54]; the leukocyte immunoglobulin like receptor B4 (*Lilrb*4a), alias ILT3, thought to control inflammatory responses and limit auto-reactivity through Treg enhancement [55]; the major histocompatibility complex (class II) molecule *Cd*74, an important regulator of immunity and inflammation with an impact on the cell endosomal compartment [56]; and the lysozyme 2 (*Lyz*2) gene involved in activities such as reducing the presence of proinflammatory cytokines (TNF-α, IL-6, INF-γ, IL-8 and IL-17) while increasing levels of anti-inflammatory cytokines (IL-4 and TGF-β) [57]; by the cost-effective alternative approach RT-qPCR (real time polymerase chain reaction after retro-transcription).

This entitles a rather easy implementation of molecular marker monitorization in follow-up studies, allowing the translation of these results to the clinic. Moreover, all these molecular changes appeared unaffected in low load treatments (1.4 N) and upregulated by medium load treatments (4.5 N), indicating that a minimum pressure is required to register the effect. High-load treatments (11 N) showed extracellular edema and different patterns that fit with induced muscle damage. 

In rats, CD68^+^ and CD163^+^ macrophage subpopulations correspond to pro-inflammatory (M1) and anti-inflammatory (M2) subtypes, formerly known as ED1^+^ and ED2^+^, respectively. Macrophages expressing pro-inflammatory M1 markers preferentially associate with proliferating muscle-precursor satellite cells, whereas macrophages mainly express anti-inflammatory M2 phenotype on myogenic differentiation stages [58]. This, together with the fact that CCR2 null mice display retarded inflammatory process and deficient muscle regeneration characterized by poor macrophage recruitment and adipocyte infiltration [53,54,59], suggests that, in fact, the CCL treatment studied by Dr. Dupont-Versteegden´s group [40] induces muscle regeneration.

Another study in C57/BL6 mice reports that massage-like stroking boosts thymic and splenic T cell numbers with statistical significant changes in double positive CD4^+^ CD8^+^ T-cells, as well as in single positive CD4^+^ or CD8^+^ cells. This increase in cell counts was correlated with decreased noradrenaline levels and reduced noradrenergic nerve fibers of the thymus and spleen, possibly mediated by chatecholamines, even partially reverting the immunosuppressive effect of hydrocortisone on CD4^+^ CD8^+^ T cells [41], indicating that massage may support recovery of immune function in individuals affected with immunodepression. 

The group led by Yokota, on another side, used a commercial knee electro-mechanical loading system (ElectroForce 3100, Bose Corporation, Eden Praire, MN, USA) which applies lateral loads to the knee to induce anabolic responses in the skeleton [60,61] to study the effects of this treatment in rat brains [42]. The rationale behind their hypothesis derived from the observation that physical activities, regularly involving application of a mechanical load on the skeleton, seem to have a stimulatory role in pain control, neural regeneration and synthesis of neurotransmitters [43,62,63]. The authors show that by using RT-qPCR, western-blot and immunohistochemistry analysis, that knee loading of 1 N at 5Hz for 1500 cycles and a 5 min treadmill running (positive control) upregulated messenger RNA (mRNA) levels of tryptophan hydroxylase 2 (*Tph*2) in the raphe nuclei of brain stem, the site of serotonin synthesis in the brain, in reference to sham load and 90 min tail suspension (stressed negative control) [42]. In addition, these authors showed that the mRNAs encoding two transcription factors of the *Tph*2 gene (*Sim*1 and *Pet*1) were significantly upregulated by this knee-loading treatment as well [43]. Reduced serotonin or *Tph*2 expression have been linked to depression, schizophrenia, and Alzheimer’s dementia-associated neurodegeneration [64,65,66], suggesting that restoration of serotonin levels through mild knee-loading may have therapeutic effects for these disorders. 

### 2.2. Effects of MT in Muscle Regeneration

In 2016, the group led by David J. Moone evaluated the regeneration of severely injured muscle by cyclic mechanical compressions driven by the combined use of external magnets and biphasic ferrogels, as an alternative mode to delivering a variety of growth factors, such as insulin-like growth factor or IGF, fibroblast growth factor-2 or FGF-2, among others [44,67]. The study was based on the observation that skeletal muscle and satellite cells are sensitive to biophysical micro-environmental cues, such as mechanical loading and stretch-associated progenitor activation [45,68]. The treatment consisted of stimulations at 1 Hz for 5 min every 12 hr by approaching and retracting a magnet to the tibialis anterior muscle subcutaneously implanted ferrogel on a murine model of myotoxin-induced or hind limb ischemia. Damaged muscle in these models lead to substantial muscle necrosis, fibrosis and contractile function loss if left untreated. The results showed that 2 weeks after treatments, mice presented greater mean muscle fiber size than the untreated, and an approximate 3-fold increase in maximum contractile force, indicative of effective muscle regeneration. Interestingly, the effect involved only the treated extremity and led to a reduction of M1 macrophages in the tissue, suggestive of a potent immune modulatory role for cyclic mechanical compressions. This treatment induced a temporary increase in intramuscular oxygen concentration which remained elevated until stimulation ceased. However, angiogenesis remained unaffected by the treatment according to unaltered average capillary density in muscle sections and no differences were observed for the endothelial marker CD31 [44].

Also recently the group of Dupont-Vergesteegden used their CCL device (4.5 N load at 0.5 Hz frequency for 30 min every other day for four bouts during a regrowth period of eight days) on hindlimb unloaded Fischer-Brown Norway rats, finding that the CCL treatment applied induced an anabolic response in muscles helping them regrow after an atrophy-inducing event. These authors conclude that massage can be used as an intervention to aid in the regrowth of muscle lost during immobilization, thus, MT-based programs that include medium load pressure may help recover muscle mass in sedentary deconditioned individuals such as patients severely affected of FM and/or CFS/ME. Interestingly, they also found that the contralateral non-massaged limbs exhibited a comparable 17% higher muscle fiber size compared to reloading alone suggestive, as formerly observed for other markers, of a systemic effect of CCL. The authors indicate that the mechanism could, at least in part, be mediated by the presence of *Pax*7^+^ cells induced by the CCL treatment [45]. 

PAX7 expression, a satellite cell marker and transcription factor associated to muscle differentiation, is regulated by microRNA-431 in that tissue. Interestingly enough, miR-431 attenuates the muscular dystrophic phenotype in *mdx* mice (a model of Duchenne muscular dystrophy) and has been proposed as a potential therapeutic target in muscular diseases [69]. In addition, miR-431 is a key post-transcriptional regulator for axon regeneration, during neural development, for brain function, and in neurological diseases [70], although this aspect has not yet been explored in relation to MT. Also, PAX7’s function is conditioned by post-translational modifications such as SUMOylation [71]. Further work is required to more clearly understand the link between MT therapeutic effects and this molecular marker.

MicroRNAs currently comprise a collection of 4690 unique small RNA sequences (miRbase v22) of 20–24 nucleotides that work as epigenetic regulators of gene expression, mainly by inducing the degradation of their target mRNAs [72,73]. Their stability and their potential to control different targets has attracted their study as potential sensors of biological processes and, thus, as biomarkers of disease. The fact that molecular alterations precede physiological and morphological changes in the cell and that miRNAs can be accurately quantitated by relatively easy cost-effective methods should make them attractive candidates to objectively evidence the impact of MT on the treatment of FM and CFS/ME.

Along the trend of the knowledge that cells sense their physical environment and that the physical application of forces translate into changes of patterns in gene expression [51,52], those miRNAs that are mechanosensitive, meaning that their levels appear regulated by mechanical cues, have been coined as mechanomiRs [46]. Although the role of cytoskeletal proteins in force transmission and mechanotransduction is quite well established [46,74], there is a paucity of knowledge regarding mechanosensitive gene regulatory networks. 

The group of Aladin M. Boriek used the mouse *mdm* (muscular dystrophy or MD with myositis) model to identify gene regulatory networks in normal and defective organisms using an *ex-vivo* model of mechanical stretch (passive stretching of approximately 0.4 N/cm in the longitudinal or transverse direction to the muscle fibers), as that information could lead to novel therapeutic approaches for MD. Their genome-wide microarray results show a list of anisotropic regulated mecanomiRs which are interestingly grouped into clusters of bicistronic or polycistronic transcriptional units from close genomic loci (<10 kb) suggesting that these mechanomiRs may present similar or coordinated biological functions [46]. In addition, the authors also found that the stretch applied significantly altered the microRNA synthesis and processing machinery. In particular, they found that stretching upregulated the nuclear protein Drosha, the cytoplasmic factor Dicer, the microRNA export protein Exportin-5, and Argonaut proteins (1–3 and 5) both in wild type (*wt)* and *mdm* mice, while not affecting the levels of the DiGeorge syndrome chromosomal region 8 (DGCR8). Moreover, the overall levels of expression of these components of the miRNA machinery were significantly higher in *mdm* than in *wt* individuals [46], suggesting a higher sensitivity of this machinery to mechanical stress in neuromuscular disorders. 

Other authors have identified individual mechanomiRs and demonstrated their role in human disease, as it is the case of miR-146a, which regulates mechanotransduction and pressure-induced inflammation in cultured human small airway epithelium [75], miR-126, which has been linked to angiogenesis [76], the let-7 family of miRNAs associated with aging and cancer [77], whose down-regulation, together with miR-98-5p may compromise satellite cell proliferation and muscle regeneration capacity [46]. Interestingly some of these mechanomiRs are expressed in non-skeletal muscles, opening the possibility for liquid biopsy testing of patients subjected to MT. Caution in the interpretation of miRNAs levels is advised, as their regulatory function will depend on the cell target and their role is not limited to down-regulating the mRNA of target genes. 

### 2.3. MT Impact on Pain Relief

Since the first animal model of nociception was described in the 19th century [78], many interventions and strategies have been used to simulate the mechanism of injury, comprising mechanical, thermal, neuropathic, inflammatory, or other on the affected tissue. For example, neuropathic models are generated by spinal nerve ligation surgery, chronic constriction, or sciatic nerve injury, while inflammatory pain is usually reproduced by injection of different substances such as capsaicin or Freund’s complete adjuvant (CFA), or the irritant carrageenan. For a comprehensive compilation on animal models of pain, we refer the readers to the review by Gregory et al., [79]. More recently, rodent models that mimic the signs and symptoms of FM, including long lasting hyperalgesia without overt peripheral tissue damage [80] and also CFS, including mechanical allodynia and hyperalgesia without signs of inflammation and injury but activated microglia [81], have been developed. While a variety of methods such as repeated muscle insults with acid injections, depletion of biogenic amines, and stress were used for the first model, a multiple continuous stress of housed in a cage with a low level of water (1.5 cm in depth) was used for the second [80,81]. Although these models reproduce some of the FM and CFS patients´ symptoms, most likely, they do not replicate these complex diseases; thus, caution is recommended when translating findings to the clinic.

The relationship between miRNA expression profiles and chronic pain has been studied in animal models at different levels: at the peripheral sensory neuron level, with soma in the dorsal root ganglion (DRG) and their axons in the skin and other organs; at the spinal cord dorsal horn (SDH) level, where secondary neurons receiving nociceptive stimuli from the periphery send them to the brain; and at the level of different parts in the brain. 

Following this order, from peripheral perception to the brain, we should mention the study by Aldrich et al., in 2009, that used a modified version of the spinal nerve ligation (SNL) model in rats, in which only the L5 spinal nerve was ligated, finding a sensory organ-specific cluster of miRNAs including miR-96, miR-182, and miR-183 that were highly enriched in the DRG. The levels of all three miRNAs in this cluster appeared significantly reduced in injured DRG neurons. Moreover, their uniform distribution within the DRG soma of non-allodynic animals was changed in allodynics, where they preferentially localized to the periphery of neurons [82]. The redistribution of these miRNAs followed the pattern of distribution of the stress granule protein T-cell Intracellular Antigen 1 (TIA-1) and could be associated with nerve damage. Sometime later, Lin et al., confirmed that SNL-induced mechanical allodynia significantly correlates with miR-183 inhibition in DRG cells. They also showed that increased intrathecal expression a of miR-183 decreased SNL-induced upregulation of Nav1.3 and BDNF (brain-derived neurotrophic factor), interestingly associating with significant attenuation of allodynia [83]. 

In another study, Tam et al. showed that miR-143 expression levels were significantly reduced in DRGs ipsilateral to CFA injection or after nerve damage [84], coinciding with our findings that miR-143 is downregulated in the PBMCs (peripheral blood mononuclear cells) of patients of FM suffering of chronic fatigue [16]. This miRNA, however, has been reported to be upregulated in the plasma of CFS/ME patients [19]. It should be pointed out that the differences found across different pain models suggest the existence of disorder-specific miRNAs rather than common miRNA regulators of nociceptive modulation. For example, members of the miR-34 family are strongly underexpressed following neuropathic pain induction, while it appears to be highly overexpressed following bone metastatic pain induction in DRG [85,86]. Also, it has been described that the interactions between sensory neurons and non-neuronal cells such as immune cells and microglia modulate nociceptive sensitivity [87], and therefore changes in other cells of the body, such as blood cells, might be indicators of individual changes in nociceptive thresholds. In fact, even if the alteration patterns of deregulated microRNAs do not match those of the tissues affected, blood miRNA patterns might still serve as reporters of health status. This is particularly relevant, as it opens the possibility of a liquid biopsy to detect and monitor nociceptive sensitivity. 

Interestingly enough, the mechano-miR 146a that has been reported among the list of miRs that are deregulated in CFS/ME [88], appears upregulated in the synovial tissue of rheumatoid arthritis patients, in the cartilage of osteoarthritis patients, and in human monocytic cell lines after lipopolysaccharide (LPS) proinflammatory stimuli [89,90,91], while it appears to be downregulated, both in the ipsilateral DRG and at the SDH level [92]. 

Other miRNAs linked to FM and CFS/ME, in particular miR-21 and miR-223, also associate to pain in animal models [16,88,93]. While both miRs are increased in spinal cord after spinal cord injury, the second also increases in the prefrontal cortex of the brain in a model of carrageenan induced facial inflammatory pain. Importantly, the overexpression of miR-223 coincides with the peak of mechanical hyperalgesia, suggesting a role of this miR in the process [94,95,96]. Regarding deregulation of miR-21 and its connection to pain mechanisms we should point out that Simeoli et al., have recently shown that primary cultured DRG neuron cell bodies release extracellular vesicles (EVs), including exosomes, loaded with miR-21 upon capsaicin activation of TRPV1 receptors. These miR-21-loaded vesicles are readily phagocytosed by macrophages inducing a pro-inflammatory phenotype. Moreover, intrathecal delivery of an antagomir of miR-21 or its conditional deletion in sensory neurons lower neuropathic hypersensitivity and inflammatory macrophage recruitment to the DRG, indicating that the induction of miR-21 expression and its release contributes to sensory neuron-macrophage communication after peripheral nerve damage [97].

Since some of the miRNAs associated with pain initiation and maintenance have also been classified as mechanomiRs [46], it seems logical to think that MT might have an impact on their expression profiles. Perhaps it is through the regulation of mechanomiR levels that MT exerts at least some of the attributed analgesic effects [98]. 

## 3. The Rationale for Using MT to Treat FM and CFS/ME Dysfunctions

A systematic review and meta-analysis of nine RCTs, including 404 FM patients, has concluded that MT with a duration of at least five weeks has beneficial immediate effects on improving pain, anxiety and depression in these patients [35]. While some previous reviews of the effect of MT for the treatment of FM symptoms coincide with this report, by concluding that MT provides benefits to FM patients [99,100], others showed negative [101] or inconclusive [102,103] results. However many of the studies included in these reviews were only qualitative in nature, or they constituted preliminary pilot studies, including a small number of participants. Li et al., argue as a possible explanation of their positive findings that their review included a larger number of RCTs, and that their analysis contemplated subgrouping based on different durations of MT [35]. This reinforces the need for MT parameter standardization.

In addition, a systematic review and meta-analysis including 60 high-quality and seven low-quality RCTs indicates that MT effectively treats pain, and that it is also beneficial for treating anxiety in the general population [104]. Another study of the same type, including a total of 140 studies, claims that MT is the most powerful method for reducing DOMS (delayed onset muscle soreness) and fatigue after exercise, compared to a compression garment, electrostimulation, stretching, immersion or cryotherapy [105]. The authors observed a moderate decrease in the muscle damage marker creatine kinase (CK) and in the inflammation markers interleukin-6 (IL-6) and C-reactive protein. 

On another side, the analysis of biopsied quadriceps (vastus lateralis) from 11 male volunteers showed that MT reduces inflammation after exercise-induced muscle damage by activating the mechanotransduction signaling pathways focal adhesion kinase or FAK, and extracellular signal regulated kinase (ERK) 1/2, inducing mitochondria biogenesis signaling, and by diminishing the levels of the inflammatory cytokines TNF-α and IL-6 and the stress factor HSP27 [106], changes that could benefit FM and CFS/ME patients [107,108]. Combinations of MT and stretching have also been studied, showing a significant reduction in fatigue with faster and shorter reduction of fatigue in females [109].

Among the models that have been developed to explain the physiopathology of FM and CFS/ME, one, at least partly, seems to set some basis for a potential impact of MT treatments in, not only alleviating symptoms, but also in delaying the progress of the disease: the neuromuscular strain model described by Rowe et al. [110]. These authors propose that “neuromuscular strain”, defined as an adverse neural tension and strain in muscles, fascia, and other soft tissues, acts as a contributor to cognitive and other symptoms in CFS [111]. If the ability of the nervous system to undergo accommodative changes in length as a response to the habitual limb and trunk movements is impaired by the restriction of movements, the mechanical tension within nerves increases, leading to neurodynamic dysfunction, these authors argue. This dysfunction contributes to pain and other symptoms that CFS patients present with, by processes of mechanical sensitization, altered nociceptive signaling, and reduced intra-neural blood flow, adverse patterns of muscle force and contraction, plus inflammatory neuropeptide release. Supportive of this model is the preliminary data obtained by these same authors from a longitudinal study of two years of duration in 55 CFS patients, showing that neuromuscular restrictions are common in CFS [110]. In addition, they show that longitudinal strain applied to nerves and soft tissues of the lower limbs is capable of increasing symptom intensity in individuals with CFS [111], supporting their model. If the neuromuscular strains are left untreated, the individual will adapt to increased symptom burden, leading to increased impairment and central sensitization. The interventions recommended by these authors to prevent symptom aggravation are MT, exercise-based approaches, or alternative therapies such as yoga or Tai Chi. In fact, they report the clinical improvement of patients by MT approaches [110]. This model seems to indicate that an action to release neural tensions at early stages of the disease might be most effective.

When MT is applied to soft and connective tissues, local biochemical changes (lactic acid, adenosine triphosphate or ATP, and creatine phosphate or CP) occur, and local muscle blood and lymph circulation increase. As result, local nociceptive and inflammatory mediators may be reabsorbed [112]. Other types of compressive treatments, such as neuromuscular taping, which also increase lymphatic and vascular flow, strengthening weakened muscles, led to the identification of a panel of miRNAs that changed with treatment in a multiple sclerosis (MS) patient [113]. Interestingly enough, some of these miRNAs have been shown to appear deregulated, both in FM [16,93,114] and in CFS/ME patients [88] suggesting that compressive treatments might provide therapeutic benefits for them as well.

On another side, MT improves pain by the modulation of serotonin levels in patients with CFS/ME and FM [50,115], changing neural activity at the segmental level, an area that is responsible for mood and pain perception [116]. MT delivery could result in the reduction of the H-reflex with pressures as low as 1.25 kPa, which would be desirable for FM patients, as spinal hyper-excitability is associated with a variety of chronic pain syndromes [117,118]. Also, myofascial stretching transduces into electrophysiological activity, which could reduce pain and other symptoms through myofascial communication, and through afferent neural pathways that modulate the subcortical nuclei and limbic system in the brain [119]. MT reduces circulating cortisol levels [50] and increases β-endorphin levels following a 30 min massage [120], which could explain reductions in perceived fatigue following MT. It will be desirable to correlate MT outcomes in patients with the markers that have been identified to associate with particular parameters of pressure and/or stretching in animal models, which will pave the way towards disease-oriented MT treatments. However, the biomarker information from animal models is presently scarce and they are yet to be evaluated by RCTs.

In this sense, Roberts has checked not only the magnitude of loading in MT but also the pattern applied. In particular, he tested three different levels of pressure in two different serial orders or patterns (increasing and decreasing) by using electromyography to measure muscle activity, finding that the physiological response of the muscle, in fact, depends on the pattern of applied pressures during massage, as only the decreasing pattern altered the electromyographic recordings [121]. This finding, according to the author, is consistent with a mechanism by which light or moderate pressure massage may reduce the gain of spinal nociceptive reflexes, which are typically elevated in chronic pain syndromes.

With respect to musculoskeletal deconditioning or muscle atrophy associated to long periods of inactivity which often affects CFS/ME and some FM patients, especially in severe cases, Rullman et al., have shown, by replicating microgravity unloading through 21 days of sustained bedrest and hypoxia, that the majority of miRNAs that become deregulated belong to miRNA families that respond to mechanical loads (mechano-miRs) [46,122]. Interestingly enough, some of these miRs associating to microgravity unloading appear to be deregulated in FM and CFS/ME patients [16,17,18], suggesting that compressive MT may provide therapeutic effects by restoring miRNA levels in muscles.

In addition to the compressive component of MT that induces changes in mechano-sensitive receptors, mechanomiRs and other molecules that are sensitive to this physical input, or effects in the immune and sensorial systems, MT also inherently contains an emotional component that is transferred to the patient through mental relaxation by the sense of touch. In fact, a positive emotional stimulus, such as watching humor videos, has been reported to increase NK cytotoxic activity only 12 hr after exposure [123]. In another study, a program of eight weeks consisting of 20–30 min/day meditation at home, six days/week, for mindfulness-based stress reduction (MBSR), showed increased killing activity of NK cells only in subjects who reported an improvement [124]. As stated formerly, animal stroking presented different responses to those elicited only by compression [41]. Also, MT of preterm newborn infants involving low pressures induces a positive effect in weight gain and an increase in vagal tone [125]. These observations indicate that MT protocols may have different levels of effects on different individuals, and are context-dependent (operator and environment), leading to heterogeneous responses, a limitation for experimental reproducibility that appears difficult to control.

On another side, it is important to note that the state of central sensitivity defined for FM, and for the threshold of hyperalgesia or allodynia for patients in general (i.e., pain induced by touch or massage) may impose limitations to MT therapeutics, as certain forces seem to be required to induce molecular changes, and therefore benefits, in animal models [40,45,126]. In fact, by assaying manual forces of 0.76 to 4.54 N/cm to obtain hypoalgesic effects McLean et al. concluded that the level of applied force was critical for pain relief setting its value beyond 1.9 N/cm (*p* = 0.014) for lateral glide mobilization. The intensity of therapeutic forces might be perceived by FM patients as an unbearable pain, restricting its use. However, as the compressing effects have been shown to be systemic, impacting contralateral untreated limbs, at least in animals [40,45], MT could be concentrated to particular low-pain areas of the body and yet obtain overall pain-reducing benefits in patients. 

## 4. Future Directions

The design of effective reproducible MT treatments, in general, relies on the standardization of protocols by rigorously defining compressive and stretching forces, the extension of the area treated, and frequency of applied movements. The parameters to be set in the protocol should be justified with controlled findings. In this respect, animal experimentation seems to be fundamental in determining the physiological and molecular changes that associate with treatments. With an interest in identifying the potential benefits of MT for the treatment of FM and CFS/ME, a review of the impact that MT may have on muscle regeneration, so that deconditioned or atrophied muscles recover, on pain relief and on the immune and neural systems, is presented in Section 2 of this manuscript. The evidence obtained from animal experimentation using mimetic devices is considered to be valuable but incomplete. Although the response to MT maneuvers at the molecular level is clear, for example, the tolerance associated marker ILT3, which could benefit autoimmune diseases [55], appears to be induced by medium load pressure treatments [40], and many miRNAs respond to certain compressive loads [46], the current paucity of information limits the potential value of adapting MT to particular health problems at present. 

**Figure 1:**
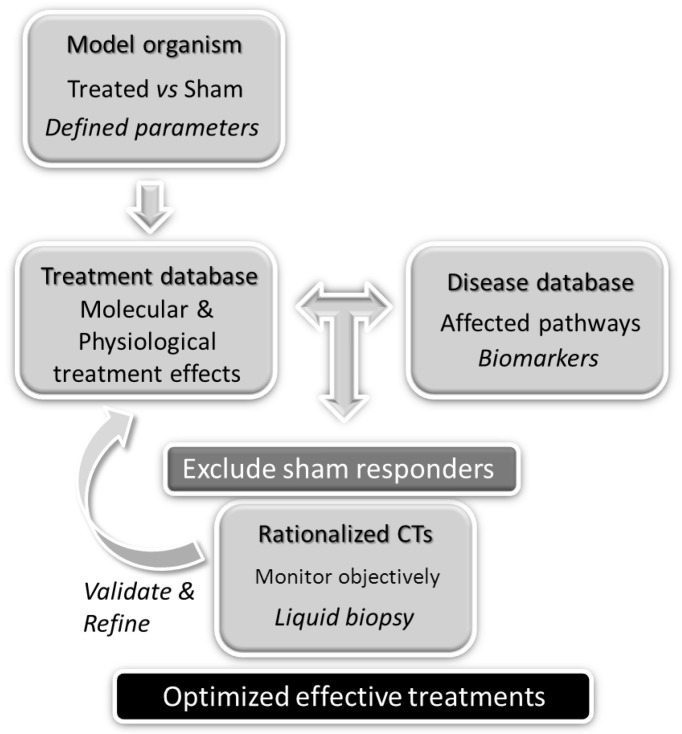
MPTA to define and standardize effective disease-tailored physiotherapy protocols flowchart.

However, this aspect may soon change. In fact, as of 30 June 2018, the number of studies registered in PubMed containing “mRNA” and “physiotherapy” terms was 764 versus, only 63 for “miRNA” and “physiotherapy” key search words. For the first group, the trend shows a marked increase in the past decade (2009–2017), with 71% of the studies found vs only 19% for the previous decade (1998–2008), while in the second group, the oldest publication date was 2008, reflecting a growing interest in evaluating the effects that physiotherapy induces in organisms at the molecular level. It will be through the building of databases nurtured with molecular and physiological observations in animals and other experimentation models that researchers will be able to design rationalized disease-focused MT-based CTs. The results of CTs importantly should be used for the validation and refinement of initial protocols in continuation CTs to finally unravel optimized effective physiotherapy-based therapeutic programs for particular health problems. Below we show a flow chart for a proposed set of future actions that require efforts from multidisciplinary teams, leading to the design of reproducible standardized effective physiotherapy treatments to different disease states by MPTA (Figure 1). 

The potential limitation of miRNA profiles as reporters of disease or as biomarkers of response to treatments should be noted. Future studies might identify other non-coding RNAs such as circular RNAs, piwiRNAs, small nucleolar RNAs or long non-coding RNAs, as well as mRNA or alternative splicing profiles that are associated with particular disease conditions, providing a more complete picture of the tissues´ physiologic state.

A similar approach to miRDDCR (a miRNA-based method to comprehensively infer drug-disease causal relationships) [127] could be structured to infer the MT–disease relationship, regardless of biomarker-disease relationship. As molecular biomarkers of FM and CFS/ME become available and validated, the selection of molecular determinants to monitor effects of MT on these patients will be facilitated. The fact that undamaged muscle tissue responds to a determined physiotherapy program with particular gene expression profiles does not guarantee that damaged or sick tissue will offer an equivalent response. For this reason, it is necessary that the evaluation of a treatment includes animal disease models that faithfully replicate the disease. Despite the lack of validated biomarkers for FM and CFS/ME, a few animal models have been developed [80,81], which may be used for initial comparisons.

Some MT-based clinical treatments, as it is the case of deep tissue or cross-friction massages, utilize high force to induce transient local inflammation, with the final goal of promoting repair and regeneration [128]. Although a benefit from this approach cannot be completely discarded at this point, a preferential exploration of medium load-based MT protocols is recommended for the treatment of FM and CFS/ME, with the intention of minimizing patient discomfort while providing health improvements. Massages with soft to moderate pressures, in addition, avoid fatigue after treatment. 

An important limitation to be minimized in the design of reproducible optimized standardized MT-protocols based on defined pressure and stretch intensities is the inherent affective or emotional response associated with this type of treatment. Responders to these affective cues could be controlled by applying MT protocols below threshold levels of mechanical response (sham treatments). Placebo responders will be excluded in CTs that are MPTA-based, in an attempt to isolate response to mechanical cues from affective responses (see Figure 1). 

As a way to monitor MT success towards setting the criteria for protocol optimization in CTs (validation and refinements steps; Figure 1), the concomitant health status of patients with treatment should be evaluated. It would be very helpful for this purpose to count with methods that are minimally invasive at the time that are informative and sensitive. These demands may, perhaps, be fulfilled by a liquid biopsy approach, which only requires a small amount of blood or other body fluids to readily assess changes in biomarker levels. In the case of FM and CFS/ME, which are complex diseases affecting various tissues and systems, an advantageous fluid fraction could correspond to EVs.

EVs are a mixture of vesicles with different functions secreted by all cell types. Among them, a particular set of vesicles that present with certain markers, and which generate from multivesicular bodies in the cell: the exosomes have attracted special attention for their intercellular communication functions [129]. By directional packaging of certain molecules, particularly miRNAs, these exosomes have been shown to spread and maintain disease [129,130]. The fact that EVs are released from all tissues into body fluids provides the advantage that their analysis will inform of the status of organs and tissues, potentially replacing in the future the needs of traditional invasive solid tissue biopsies. 

Other assays in body fluids not involving EV isolation are also available; for example, in a study by Arroyo-Morales et al., saliva IgA levels were used to monitor the effects of a 40 min myofascial induction by MT after exercising in healthy individuals (*N* = 60) [131]. In fact, saliva is acquiring importance as a non-invasive method for the diagnosis, prediction, and progression of several diseases [132], and it could provide an easy way to monitor the effectiveness of physiotherapy protocols in the future.

## 5. Conclusions 

In summary, we can conclude that there is an urgency to standardize, control, and optimize MT, and physiotherapy protocols in general, as the conflictive results that have been frequently found in the literature may arise from subjective components and the lack of precise parameter definition in these procedures. Gene expression information in relation to defined MT parameters could serve as guidelines for an adequate design of MT therapeutic protocols to be tested and refined through CTs. 

The potential of microRNAs and particularly mechanomiR profiles as an approach to monitor MT treatments has been evidenced here. A comparison of results from studies in animal models and MT mimetic devices, together with FM and CFS/ME patient dysfunctions, points to plausible benefits of MT treatments for these patients. Additionally, MT offers a safe alternative to physical exercise, provided that hyperalgesia and allodynia permits the application of effective pressures or stretching forces. However, a more complete view of molecular patterns associated with both disease and particularly MT protocols, are required to ensure the development of effective and safe MT-based treatments.

## Figures and Tables

**Table 1:** Summary of studies showing relevant preclinical data for initial MPTA (molecular physiotherapy approach) MT-based therapeutics.

Organism	Treatment	Parameter	Markers	Tissues/Cells Affected	Contral.	Cites
*Rattus norvegicus*	CCL mimetic device	Pressure	CCR2, ILT3, CD74, LYZ2/CD68 & CD163	Immune/skeletal muscle	Yes	[40]
*Mus musculus*	Massage-like stroking	Massage	T cell numbers/noradrenaline levels	Immune/endocrine	Unknown	[41]
*Rattus norvegicus*	Electro-mechanical loading system	Knee loading	*Tph*2, *Sim*1, *Pet*1	Brain stem	Unknown	[42,43]
*Mus musculus*	Ferrogels driven by external magnets	Pressure	Intramuscular [O_2_]	Immune/skeletal muscle	No	[44]
*Rattus norvegicus*	CCL mimetic device	Pressure	Anabolism/*Pax*7	Skeletal muscle	Yes	[45]
*Mus musculus* (*mdm*)	Ex vivo mechanical stretch	Stretching	MechanomiRs/microRNA machinery	Skeletal muscle	Unknown	[46]

(“CCL” stands for cyclic compressive loading “Contral.” refers to contralateral effects of treatments).

## Abbreviations

MT	Manual Therapy
FM	Fibromyalgia
CFS/ME	Chronic Fatigue Syndrome/Myalgic Encephalomyelitis
MPTA	Molecular Physiotherapy Approach
CCL	Cyclic Compressive Loading
CT	Clinical Trial
miR	microRNA
EV	Extracellular Vesicle
ICD	International Classification of Diseases
PBMC	Peripheral Blood Mononuclear Cells
DRG	Dorsal Root Ganglion
SDH	Spinal cord Dorsal Horn
